# Neonicotinoid Seed Treatments Have Significant Non-target Effects on Phyllosphere and Soil Bacterial Communities

**DOI:** 10.3389/fmicb.2020.619827

**Published:** 2021-01-13

**Authors:** Mona Parizadeh, Benjamin Mimee, Steven W. Kembel

**Affiliations:** ^1^Agriculture and Agri-Food Canada, Saint-Jean-sur-Richelieu, Quebec, QC, Canada; ^2^Département des sciences biologiques, Université du Québec à Montréal, Montréal, QC, Canada

**Keywords:** bacterial community structure, bacterial diversity, host-microbe interactions, neonicotinoid seed treatment, pesticide non-target effects, phyllosphere, soil bacterial communities, temporal variation

## Abstract

The phyllosphere and soil are dynamic habitats for microbial communities. Non-pathogenic microbiota, including leaf and soil beneficial bacteria, plays a crucial role in plant growth and health, as well as in soil fertility and organic matter production. In sustainable agriculture, it is important to understand the composition of these bacterial communities, their changes in response to disturbances, and their resilience to agricultural practices. Widespread pesticide application may have had non-target impacts on these beneficial microorganisms. Neonicotinoids are a family of systemic insecticides being vastly used to control soil and foliar pests in recent decades. A few studies have demonstrated the long-term and non-target effects of neonicotinoids on agroecosystem microbiota, but the generality of these findings remains unclear. In this study, we used 16S rRNA gene amplicon sequencing to characterize the effects of neonicotinoid seed treatment on soil and phyllosphere bacterial community diversity, composition and temporal dynamics in a 3-year soybean/corn rotation in Quebec, Canada. We found that habitat, host species and time are stronger drivers of variation in bacterial composition than neonicotinoid application. They, respectively, explained 37.3, 3.2, and 2.9% of the community variation. However, neonicotinoids did have an impact on bacterial community structure, especially on the taxonomic composition of soil communities (2.6%) and over time (2.4%). They also caused a decrease in soil alpha diversity in the middle of the growing season. While the neonicotinoid treatment favored some bacterial genera known as neonicotinoid biodegraders, there was a decline in the relative abundance of some potentially beneficial soil bacteria in response to the pesticide application. Some of these bacteria, such as the plant growth-promoting rhizobacteria and the bacteria involved in the nitrogen cycle, are vital for plant growth and improve soil fertility. Overall, our results indicate that neonicotinoids have non-target effects on phyllosphere and soil bacterial communities in a soybean-corn agroecosystem. Exploring the interactions among bacteria and other organisms, as well as the bacterial functional responses to the pesticide treatment, may enhance our understanding of these non-target effects and help us adapt agricultural practices to control these impacts.

## Introduction

The phyllosphere (the aerial surfaces of plants including leaves) and soil are colonized by microbial communities (microbiota), which are of great importance in the regulation of host and ecosystem function. These microbial communities, including beneficial bacteria, play a crucial role in plant growth promotion, decomposition and health control (Vorholt, 2012), as well as in soil fertility, nitrogen fixation, and organic matter production (Doran and Zeiss, 2000; Garbeva et al., 2004). Previous studies have shown that the taxonomic composition of phyllosphere bacteria is associated with host plant species identity (Whipps et al., 2008; Knief et al., 2010; Kembel et al., 2014) and changes predictably during the growing season and as plant ages (Redford and Fierer, 2009; Sugiyama et al., 2014; Wagner et al., 2016). Host species were found to be a more important driver of variation in phyllosphere bacterial communities than time (Laforest-Lapointe et al., 2016b). Other studies on the composition of soil bacteria have associated community variations with host plant species and growth stage (Wieland et al., 2001), site (Clairmont et al., 2019) and time (Tarlera et al., 2008; Hannula et al., 2019). Host species were also shown to be a stronger driver of variation in soil bacterial communities than host plant growth stage and development time (Wieland et al., 2001).

Bacterial succession refers to the bacterial community variation patterns over time and in response to environmental changes and disturbances (Redford and Fierer, 2009). Comparing temporal versus spatial variation in bacterial community structure, the effects of time (seasonal variability) on bacterial communities is often higher than habitat impacts (Samaritani et al., 2017). Environmental disturbances and perturbations (such as cultivation methods, drought, climate change, and pesticide treatments) can also alter the bacterial community structure and composition (Schimel et al., 2007; Itoh et al., 2014). During the succession process, some bacterial communities may survive by modifying their habitat, increasing their abundance or becoming more resistant or resilient to disturbances (Schimel et al., 2007; Fierer et al., 2010). Hence, if a disturbance is persistent, it can cause long-term changes in bacterial community structure and affect bacterial succession (Fierer et al., 2010). During the last decades, the widespread application of chemical pesticides in agro-ecosystems has influenced many non-target species and their succession patterns (Itoh et al., 2014; Rodríguez-Valdecantos et al., 2017). Pesticides can change the interaction between plants and some bacteria, such as nitrogen-fixing rhizobacteria, which may lead to the inhibition of nitrogen fixation (Fox et al., 2007; Lo, 2010). They can also affect soil fertility and quality by impacting soil bacterial diversity and function and altering their nitrification, denitrification and mineralization of organic matter (Hussain et al., 2009). In this study, to assess the effects of pesticides on the phyllosphere and soil bacterial community structure and intra- and inter-annual succession, we have focused on a class of the most widely used insecticide pesticides, neonicotinoids.

Neonicotinoids (also known as neonics) are a family of systemic and neuro-active insecticides, chemically similar to nicotine, introduced in the late 1980s (Kagabu, 1996; Tomizawa and Casida, 2005). Like nicotine, they interrupt neural transmission in the nervous system by binding to the nicotinic acetylcholine receptors (nAChRs). Because of the fundamental distinctions between the nAChRs of invertebrates and vertebrates, neonicotinoids are selectively more toxic to invertebrates, like insects (Tomizawa et al., 1999; Tomizawa and Casida, 2003). In North America, neonicotinoids have mostly been used as seed treatments to control a variety of foliar and soil early-season insect pests in corn, soybean, wheat and other important crops (Elbert et al., 2008; Samson-Robert et al., 2014; Douglas and Tooker, 2015). These treatments are most widely applied prophylactically, without any information on the actual presence of the targeted pests. Hence, previous studies have indicated that neonicotinoids often have no significant impact on crop yield (Cox and Cherney, 2011; Reisig et al., 2012; Penn and Dale, 2017; Alford and Krupke, 2018). A recent study that has extensively evaluated yield variations in response to neonicotinoid seed treatment with regards to the abundance and incidence of pest populations has reported that there is no significant difference in crop yield when pest pressure is low, which was the case in most of the sites under study (Labrie et al., 2020). The neonicotinoid compounds are tiny molecules and are highly soluble in water (Bonmatin et al., 2015). Given their systemic nature, plants take them up from the seed covering and translocate them to different tissues and products, including nectar, guttation and pollen (Sur and Stork, 2003; Bonmatin et al., 2005; Girolami et al., 2009). Neonicotinoids may remain active from 20–30 days in soybean (Myers and Hill, 2014) and corn (Alford and Krupke, 2017) to 200 days in winter wheat (Zhang et al., 2016). Plants only absorb about 20% of the seed covering (Sur and Stork, 2003; Alford and Krupke, 2017). The rest of the pesticide persists in soil for up to 3 years, depending on its active ingredient and the soil properties (e.g., soil type, organic matter content and pH) (Goulson, 2013; Bonmatin et al., 2015). During the last decades, many questions have been raised about the potential impacts of the widespread and prophylactic (Goulson, 2013; Labrie et al., 2020) use of neonicotinoids on non-target organisms. Past studies have shown some negative effects of neonicotinoids on agriculturally beneficial organisms, including beneficial soil invertebrates like earthworms (Pisa et al., 2015), and insect pollinators, particularly honeybees (Iwasa et al., 2004; Samson-Robert et al., 2014, 2017; Sanchez-Bayo and Goka, 2014; Bonmatin et al., 2015). Although neonicotinoids target organisms that possess a nervous system and the nAChRs, some studies have reported that they have non-target impacts on the functions and structure of microbial communities, such as fungal (Moulas et al., 2013) and bacterial structure, abundance and community composition in phyllosphere (Zhang et al., 2008, 2009; Moulas et al., 2013) and soil (Cycoń et al., 2013; Yu et al., 2020). Previous biochemical or culture-based microbiological studies have also confirmed the effects of these insecticides on bacterial respiration, phosphatase activity, and other enzyme activities, including ammonification, nitrification, and denitrification (Singh and Singh, 2006; Ahemad and Khan, 2012; Cycoń and Piotrowska-Seget, 2015; Filimon et al., 2015).

Soybean and corn are two important agricultural crops and are among those that are typically treated by pesticides, including neonicotinoids. In this study, we aim to (1) characterize the drivers of variation in bacterial community structure of soybean and corn phyllosphere and soil and (2) identify the responses of bacterial community composition variation and diversity to neonicotinoid seed treatment in a 3-year soybean/corn rotation. We hypothesized that (1) habitat, host species and time will all contribute to variation in bacterial community composition and diversity, and (2) neonicotinoid seed treatment will cause a shift in the bacterial community composition and a decrease in bacterial diversity of both phyllosphere and soil. We address these objectives and hypotheses by quantifying bacterial community structure using bacterial 16S rRNA gene amplicon sequencing in soybean and corn phyllosphere and soil samples collected over 3 years in Quebec, Canada.

## Materials and Methods

### Study Site

We cultivated a 3-year rotation of soybean (2016 and 2018) and corn (2017) on the Agriculture and Agri-Food Canada experimental farm in L’Acadie (ACA) (45°17′38.0″N; 73°20′58.0″W), Quebec, Canada. L’Acadie is located in Canadian hardiness zone 5a. The region is characterized by having a clay loam soil type and a temperate climate. In mid-May of each year, we sowed soybean or corn on a 100 m × 30 m field, previously a meadow, that had not received neonicotinoid application during the 3 years preceding the experiment. Four replicates of each non-neonicotinoid (control) and neonicotinoid-treated plots (100 m × 3 m) were established alternately and consisted of four rows each. Two extra neonicotinoid-treated plots surrounded the experimental field. Soybean and corn seeds were coated with three fungicides (difenoconazole, metalaxyl-M, and sedaxane) in both control and treated plots. The neonicotinoid-treated seeds were also covered by thiamethoxam at 0.25 mg/seed. The fields were under no-till farming, and glyphosate was applied twice during each growing season (before seeding and one month after it) to control weeds. The corn field was also fertilized with 400 kg/ha NPK (15-15-15) before seeding and 222 kg/ha N (27.5%) 1 month after seeding. Soil physicochemical properties (e.g., pH, etc.) were constant across the experimental field and did not differ between the growing seasons (Supplementary Table 1).

### Sample Collection

To study the phyllosphere bacteria (the bacteria collected from the leaf surface in our case), each year we collected 48 samples (two samples per plot at three sampling times during the growing seasons), for a total of 144 samples. The three annual sampling occasions happened in July, August and September. We sampled 50–100 g of healthy mature middle leaves of 6–10 close plants from the two middle rows of each plot. We then stored each sample in a sterile plastic bag and transferred it to the laboratory in a cooler, surrounded by ice packs. We immediately collected the bacterial cells from the leaves by washing them in a 0.85% saline solution and agitating the solution using a stomacher at 250 rpm for 30 s. We then transferred the solutions to 50-ml tubes, centrifuged them at 4,000 *g* for 20 min and discarded the supernatants. We kept the remaining pellets at −80°C until use.

To study the soil bacteria, we sampled bulk soil (soil that does not adhere to plant roots) from the upper 12–15 cm layer of soil with a corer (2 cm in diameter) from the soil around the same plants that we sampled for the phyllosphere. For each soil sample, we collected soil from six different spots, in a zigzag pattern and at 10 cm from the plants, and then mixed and pooled them into one 400–500 g sample (Sugiyama et al., 2014; Gagic et al., 2017). We transferred samples to the laboratory in a cooler and stored at −80°C until use. Each year, we collected 48 soil samples (two samples per plot at the same three sampling times as phyllosphere), for a total of 144 samples.

### DNA Extraction

We extracted DNA from the samples of phyllosphere (pellets containing bacterial cells) and soil (directly) using MoBio PowerSoil DNA isolation kit (QIAGEN). Considering the high amount of material to be extracted from each soil sample, we extracted DNA twice, each time from 0.5 g of the same sample, and pooled the extractions together in order to better capture soil bacterial community variation. The rest of the extraction was performed according to the manufacturer’s instructions. Then, we measured the concentration and quality of the extracted DNA using Qubit (Thermo Fisher Scientific) and Nanodrop (Thermo Fisher Scientific) prior to storing them at −80°C.

### Bacterial DNA Amplification

Following previously described protocols (Kembel et al., 2014; Laforest-Lapointe et al., 2016b; Kim et al., 2018), we amplified the V5–V6 hypervariable regions of the bacterial 16S rRNA gene, using chloroplast-excluding primers [16S primers 799F-1115R (Chelius and Triplett, 2001; Redford et al., 2010)]. We added variable length barcodes and Illumina adaptor sequence to the 5′ end of the primers. Each PCR reaction (25 μL) contained 1 μL of genomic DNA (1:10 dilution for soil samples), 5 μL 5xHF buffer (Thermo Scientific), 0.5 μL dNTPs (10 mM each), 0.75 μL DMSO, 0.25 μL Phusion Hot Start II polymerase (Thermo Scientific), 1.0 μL of each primer (5 μM), and 15.50 μL double-distilled water. We amplified the bacterial DNA in an Agilent SureCycler 8,800 using the following conditions: 98°C for 30 s, 35 cycles of 98°C, 15-s denaturation; 64°C, 30-s annealing, and 72°C, 30-s elongation; followed by a final elongation at 72°C, 10 min. All samples were distributed randomly into several 96-well PCR plates for DNA amplification. Each PCR plate contained one positive and one negative control. Each positive control included *Clavibacter michiganensis*, *Pectobacterium* sp., *E. coli* DHS alpha, *Pantoea stewartii* and *Xanthomonas* sp., while the negative controls were nuclease-free, DEPC-treated and autoclaved water. We also had negative controls of the sampling plastic bags, tubes and the extraction kit. All PCR products were electrophoresed on a 2% agarose gel in 1X TAE buffer, stained with AMRESCO’s EZ-Vision dye as loading buffer (VWR Life Science), and visualized by G:BOX gel doc (Syngene).

### Normalization, Library Preparation and Sequencing

All PCR products were normalized using SequalPrep PCR Normalization kit (Thermo Fisher Scientific). One library per PCR plate was prepared by pooling all the amplified and normalized DNA. The concentration of each library was determined using Qubit. For each sequencing run, an equimolar concentration of each library was pooled and purified using Ampure XP (Beckman Coulter by Thermo Fisher Scientific), according to the manufacturer’s protocol. We used Qubit and Bioanalyzer DNA analysis kit (Agilent) to verify the final concentration and quality of the purified DNA. According to MiSeq Illumina guidance, the 4 nM DNA was denatured using NaOH 0.2 N and then diluted to a 14 pM library. Then, it was PE (paired-end) sequenced on Illumina MiSeq (2 × 300 bp), using a 600-cycle MiSeq reagent kit v3, at Agriculture and Agri-Food Canada.

### Bioinformatic Analyses

We used BBDuk^1^ to remove Illumina adapters. We also removed barcodes and primers and then demultiplexed the Illumina reads. Afterward, we applied DADA2 v1.12.1 (Callahan et al., 2016) to remove low-quality sequences, correct the Illumina-sequencing amplicon errors, merge paired-end sequences, eliminate chimeric sequences, and identify amplicon sequence variants (ASVs). We used default parameter settings for all functions except for the following functions: (i) in filterAndTrim function, we removed all the sequences with fewer than 50 nucleotides (minLen = 50, instead of 20), (ii) in dada function, we set the algorithm to perform pseudo-pooling between samples, and (iii) in mergePairs, we set a minimum overlap length of 10 (minOverlap = 10, instead of 12) in order to merge the forward and reverse reads. We finally used the RDP naive Bayesian classifier method implemented in DADA2 with the SILVA 132 rRNA database (Quast et al., 2013; Yilmaz et al., 2014) to annotate the taxonomic identity of ASVs.

#### Sample Quality Control, Decontamination and Rarefaction

**FIGURE 1:**
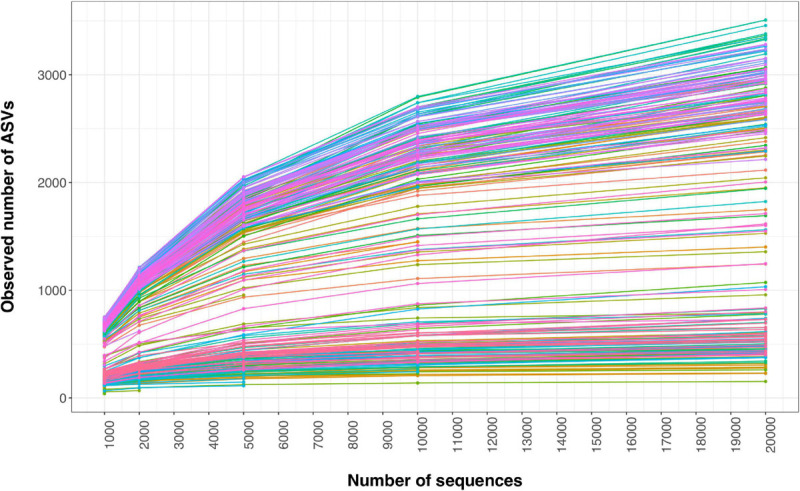
Rarefaction curves of the phyllosphere and soil bacterial ASVs. Rarefaction curves are shown for all the phyllosphere and soil samples according to the observed ASVs richness. Each line and color represent one sample. The sequencing coverage (*x*-axis: number of sequences) is 20,000 reads with cutoffs at 1,000, 2,000, 5,000, and 10,000 reads.

After verifying the presence and composition of the mock communities in the positive controls, we removed them from the dataset. To minimize sequence artifacts caused by PCR and sequencing errors (Acinas et al., 2005), which may result in spurious ASVs, we performed the following steps of quality filtering and decontamination: (1) removing ASVs that were not taxonomically annotated as belonging to a bacterial phylum (0.78% of all sequences); (2) eliminating the outlier samples (including two of the negative control samples) that had a very different composition from the other samples based on the non-metric multidimensional scaling (NMDS) on Bray-Curtis dissimilarities (Bray and Curtis, 1957); (3) filtering all the samples with less than 1,000 sequences (39 samples, including all the other negative controls, except for the phyllosphere and soil sampling bag controls); (4) removing the contaminating DNA from the bacterial communities using the prevalence method (probability threshold = 0.5) of the decontam package v1.1.2 (Davis et al., 2018) in R v4.0.0 (R Core Team, 2019), which identified 50 ASVs as contaminants based on the most prevalent ASVs in the negative controls; (5) eliminating all the ASVs recognized as chloroplasts or mitochondria (0.15%); (6) excluding the samples with low alpha diversity (Shannon richness <2, including the soil sampling bag control and one phyllosphere sample); (7) removing the rare ASVs with less than 10 reads (37% of ASVs); and (8) eliminating again the outlier samples detected in the denoised dataset (five samples, including the last remaining negative control, one phyllosphere and four soil samples), which had a highly different composition (based on NMDS on Bray-Curtis dissimilarities) or species richness (based on Shannon diversity) from the other samples of the same habitat. Finally, we selected cutoffs to rarefy samples based on inspection of rarefaction curves for phyllosphere and soil samples, choosing rarefaction cutoffs that approached saturation in the ASV rarefaction curve while keeping as many samples as possible. We first rarefied the soybean and corn phyllosphere and soil samples to 5,000 reads per sample, which excluded 12 samples that contained insufficient numbers of sequences and 699 ASVs. We then made a subset of non-treated (control) samples (119 samples and 13,042 ASVs) to study the soybean and corn phyllosphere and soil bacterial community diversity and composition. We also made a subset of phyllosphere samples (110 samples and 6,695 ASVs) to study the variations in the phyllosphere bacterial community diversity and composition in response to neonicotinoid seed treatment. Since soil samples had more sequences per sample than phyllosphere samples, we rarefied the dataset again, this time to 10,000 reads per sample, which excluded 22 samples that contained insufficient numbers of sequences and 195 ASVs. Therefore, we subset soil samples to study the effects of neonicotinoid seed treatment on their bacterial diversity and composition (132 samples and 13,137 ASVs). Overall, quality control and filtering, decontamination, and rarefaction procedures at 5,000 and 10,000 cutoffs (Figure 1), respectively, eliminated 41 and 39% of the low-quality ASVs and 20 and 23% of the samples (including all the 15 negative controls). We then analyzed these datasets using different R packages.

### Statistical Analyses

#### Characterization of Phyllosphere and Soil Bacterial Composition and Diversity

To identify the bacterial composition and diversity of the soybean and corn phyllosphere and soil, we analyzed the non-neonicotinoid treated (control) samples that were rarefied to 5,000 reads per sample. This dataset contained 119 samples (including 30 soybean and 21 corn phyllosphere samples, as well as 45 soybean and 23 corn soil samples) with an average of 1,174 ± 65.0 ASVs (mean ± SE) per sample. We conducted permutational multivariate analyses of variance (PERMANOVA) (Anderson, 2001) using the adonis2 function of the vegan package in R with 999 permutations first on the whole community dissimilarity matrix to test for the effects of habitats (phyllosphere and soil), host species (soybean and corn), time (month and year), and their interactions on the bacterial composition variation (model:. ∼ habitat ^∗^ host species ^∗^ month/year), and then on each habitat individually to test for the effects of host species, time and their interactions on the bacterial community composition (model:. ∼ host species ^∗^ month/year).

To assess the bacterial community homogeneity of each habitat and also each host species individually in phyllosphere and soil, we used a multivariate homogeneity test of groups dispersions using the betadisper function of the vegan package in R and then performed an analysis of variance (ANOVA)-like permutation test with 999 permutations to evaluate the significance of the results.

Furthermore, we used the Shannon index to estimate the soybean and corn phyllosphere and soil bacterial alpha diversity. We conducted the non-parametric Wilcoxon rank-sum test (Wilcoxon, 1945) to compare the Shannon diversity for the following groups: between phyllosphere versus soil samples, and individually in each habitat between soybean versus corn samples, among years, and among months. This test was applied to determine the statistically significant differences of the bacterial ASVs richness among the mentioned groups. We adjusted the *P*-values using Holm’s method (Holm, 1979).

To understand which families drove the variation in bacterial composition across habitats and hosts, we studied the correlations among all the bacterial families of soybean and corn phyllosphere and soil, which had an average relative abundance of more than 0.01, with their habitats and hosts. To achieve this, we used the envfit function of the vegan package in R, which computes the goodness of fit values (R^2^) and their significance (with 999 permutations) of the vectors of bacterial families relative abundance onto the principal coordinate analysis (PCoA) ordination (based on Bray-Curtis distances).

#### Effects of Neonicotinoid Seed Treatment on Bacterial Community Composition, Diversity, and Temporal Variation

To study the bacterial community variations in response to neonicotinoid seed treatment, we separately analyzed the rarefied phyllosphere (5,000 reads per sample) and soil (10,000 reads per sample) samples. The phyllosphere dataset contained 110 samples (including 67 soybean and 43 corn samples) with an average of 391.1 ± 20.3 ASVs (mean ± SE) per sample, and the soil dataset contained 132 samples (including 85 soybean and 47 corn samples) with an average of 2,257 ± 30.0 ASVs (mean ± SE) per sample. We evaluated the relationships between bacterial communities and their host species, time (year and month) and neonicotinoid seed treatment, using a PERMANOVA with 999 permutations on the community matrix (model:. ∼ host species ^∗^ year ^∗^ month ^∗^ neonicotinoid seed treatment) for each habitat individually. We also performed a PCoA (on Bray-Curtis dissimilarities) per habitat to illustrate the composition variation in the bacterial communities. Given the strong effects of host plants on the phyllosphere (Knief et al., 2010; Kembel et al., 2014; Laforest-Lapointe et al., 2016b) and soil (Wieland et al., 2001) bacterial community structure and according to our preliminary results, we also studied the soybean and corn samples individually to understand whether the impacts of neonicotinoid seed treatment on the patterns of bacterial community variation were masked by host species. Thereafter for each crop, we performed a PCoA (based on Bray-Curtis distances) and a PERMANOVA test (model:. ∼ year ^∗^ month ^∗^ neonicotinoid seed treatment) to explore the phyllosphere and soil bacterial community composition and the drivers of its variation.

We used the Shannon index to determine the phyllosphere and soil bacterial alpha diversity. Then, we conducted the non-parametric Wilcoxon rank-sum test to compare the Shannon diversity between the control versus neonicotinoid-treated samples in each habitat, as well as in soybean and corn separately for each habitat (model: Shannon ∼ neonicotinoid seed treatment). For each habitat individually, we used a linear model to evaluate the effects of neonicotinoid application on the bacterial alpha diversity across time (phyllosphere model: Shannon ∼ neonicotinoid seed treatment ^∗^ month; soil model: Shannon ∼ neonicotinoid seed treatment ^∗^ month ^∗^ year), followed by an ANOVA test to determine the significant interactions. We then used the Wilcoxon rank-sum test, in which we grouped the samples by month (phyllosphere and soil models: Shannon ∼ neonicotinoid seed treatment, group by = month) and by year (soil model: Shannon ∼ neonicotinoid seed treatment, group by = year) to identify the significance of the interactions suggested by our model. We adjusted the *P*-values using Holm’s method.

#### Effects of Neonicotinoid Seed Treatment on Bacterial Taxonomic Composition

To determine the differentially abundant ASVs and taxa between control and neonicotinoid-treated samples in each habitat, we performed a differential expression analysis of sequence data [DESeq2 (Love et al., 2014)] using the Wald significance test with a local fit type and compared the results by estimating the log2 fold changes. We analyzed the non-rarefied and non-normalized quality filtered and decontaminated bacterial phyllosphere (118 samples, including 58 control and 60 neonicotinoid-treated samples) and soil samples (137 samples, including 69 control and 68 neonicotinoid-treated samples) separately to identify the differentially abundant ASVs and taxa using the DESeq2 test. We then adjusted the *P*-values (significance cutoff of 0.05) using the Benjamini-Hochberg false-discovery rate (FDR) method (Hochberg and Benjamini, 1990) to identify the significantly differentially abundant ASVs and taxa between the control and neonicotinoid-treated samples during 3 years of rotation individually for each habitat.

## Results

### Effects of Habitat, Host Species and Time on the Phyllosphere and Soil Bacterial Communities

**FIGURE 2:**
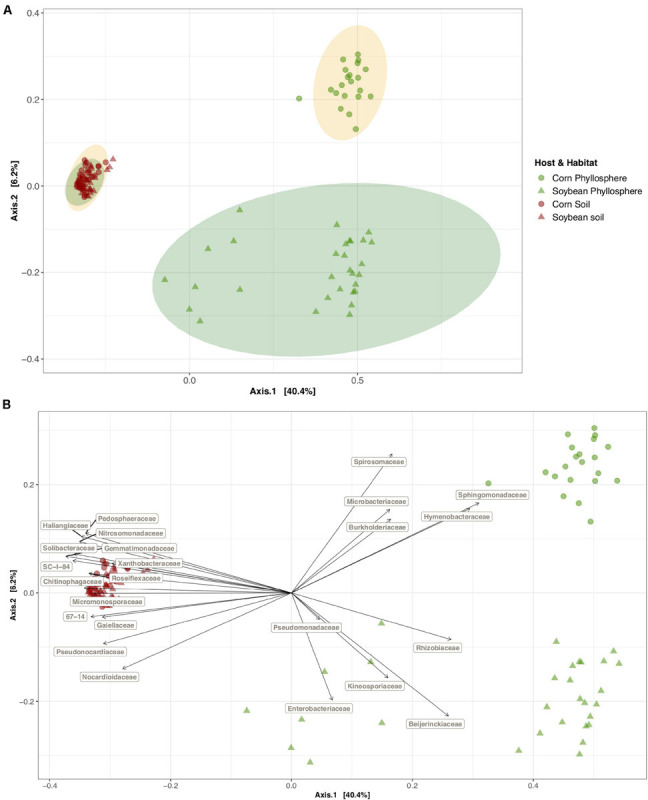
Soybean and corn phyllosphere and soil bacterial community diversity and the families who are driving this diversity pattern. Principal coordinate analysis (PCoA) on Bray-Curtis dissimilarities **(A)** of the bacterial community composition and the correlations between bacterial family abundances and different axes of the PCoA ordination **(B)** in the phyllosphere and soil bacterial communities in a 3-year soybean/corn rotation in L’Acadie, Quebec, Canada. Ordinations show that habitat (red points: soil, green points: phyllosphere) and host species (circle points: corn, triangle points: soybean) explain the bacterial community composition variations. The axes of the ordinations explain 46.6% of the variation in the bacterial community composition. Ellipses **(A)** are shaded based on host species (yellow for corn and green for soybean samples) and represent a 99% confidence level. Gray arrows **(B)** indicate the significant correlations (*P* = 0.001, except for the Pseudomonadaceae family) among the bacterial families that had an average relative abundance of more than 0.01 and their habitat and host species. Arrows directions show the correlations among habitats and host species and arrow length indicates the strength of these correlations.

In this experiment, the habitat (phyllosphere versus soil) was the strongest driver of bacterial community variation. Habitat alone explained 37.3% of the community variation, while host plant species (soybean versus corn) explained only 3.2%, and their interaction 3.7% (PERMANOVA *P* < 0.001, Table 1). Community composition was significantly more homogenous among soil samples than phyllosphere samples (average distance to median 0.42 versus 0.50, ANOVA on multivariate homogeneity of groups dispersions *F* = 24.13, *P* < 0.001) and the phyllosphere communities exhibited less variation in corn than in soybean (average distance to median 0.38 versus 0.48, ANOVA on multivariate homogeneity of groups dispersions *F* = 6.20, *P* < 0.05, Figure 2A). Bacterial alpha diversity was significantly higher in soil than in the phyllosphere (Shannon index mean ± SE 7.0 ± 0.02 versus 4.2 ± 0.10, Wilcoxon adjusted *P* < 0.0001). The relative abundance of several bacterial families was strongly associated with soil (such as Gemmatimonadaceae and Solibacteraceae), soybean phyllosphere (such as Beijerinckiaceae and Rhizobiaceae) or corn phyllosphere (such as Sphingomonadaceae and Hymenobacteraceae) (*P* < 0.001, envfit analysis of correlation between PCoA axes and variables, Figure 2B and Supplementary Table 2).

**TABLE 1:** Main drivers of the phyllosphere and soil bacterial community composition variation in a 3-year soybean/corn rotation.

	**Phyllosphere and soil**			**Phyllosphere**			**Soil**		
**Variables**	**R^2^ (%)**	***F***	**Pr(>*F*)**	**R^2^ (%)**	***F***	**Pr(>*F*)**	**R^2^ (%)**	***F***	**Pr(>*F*)**
Habitat	37.3	100.98	0.001***						
Host species	3.2	8.69	0.001***	18.6	19.62	0.001***	2.5	1.83	0.007**
Month/Year	2.9	3.93	0.001***	15.7	8.28	0.001***	4.6	1.66	0.002**
Habitat: Host species	3.7	10.07	0.001***						
Habitat: Month/Year	3.6	4.9	0.001***						
Host species: Month/Year	2.1	2.89	0.005**	11.4	6.02	0.001***	NS	NS	NS
Host species: Month: Year	2.5	2.97	0.005**	14.6	5.14	0.001***	8	1.94	0.001***
Habitat: Host species: Month	2.5	3.35	0.003**						
Habitat: Host species: Month: Year	7.2	3.26	0.001***						

PERMANOVA (Bray-Curtis dissimilarities) determines the contributions of habitat and host plant species and their interactions in the soybean and corn phyllosphere and soil bacterial composition variation in a 3-year soybean/corn rotation in L’Acadie, Quebec, Canada. (:) represents the interaction between variables. Significance levels for each variable are given by: ***P < 0.001; **P < 0.01; *P < 0.05; NS, *P* ≥ 0.05.

Time was also a significant determinant of bacterial community variation, particularly in the phyllosphere habitat. Month and year together explained 2.9% of the whole bacterial community composition variation while the interactions between time, habitat and host species explained 7.2% of the variation (PERMANOVA *P* < 0.001, Table 1). Time was a much greater driver of community composition variation in the phyllosphere than in soil (15.7% versus 4.6%, PERMANOVA *P* < 0.001, Table 1). Alpha diversity varied in time in the phyllosphere but not in soil (Table 2). This effect in the phyllosphere was especially obvious between the first and the last year of the rotation where diversity was highest in the last year (Shannon index mean ± SE, respectively, 4.0 ± 0.17 versus 4.8 ± 0.20, Wilcoxon adjusted *P* < 0.0001, Table 2) but we also observed intra-annual variation in diversity (Table 2).

**TABLE 2:** Bacterial alpha diversity explained by time (year and month) and neonicotinoid seed treatment.

**Variables**		**Phyllosphere**		**Soil**		
		**Mean ± SE**	**Adjusted *P*-value**	**Mean ± SE**	**Adjusted *P*-value**	**Subset**
Host species	Soybean	4.4 ± 0.15	NS	7.0 ± 0.03	NS	
	Corn	4.0 ± 0.12		7.0 ± 0.04		
Year	2016	4.0 ± 0.17	NS	7.0 ± 0.05	NS	
	2017	4.0 ± 0.12		7.0 ± 0.04		
	2017	4.0 ± 0.12	< 0.01**	7.0 ± 0.04	NS	
	2018	4.8 ± 0.20		7.1 ± 0.02		
	2016	4.0 ± 0.17	< 0.0001****	7.0 ± 0.05	NS	
	2018	4.8 ± 0.20		7.1 ± 0.02		
Month	July	4.6 ± 0.30	< 0.05*	7.0 ± 0.03	NS	
	August	3.8 ± 0.11		7.0 ± 0.04		
	August	3.8 ± 0.11	< 0.001***	7.0 ± 0.04	NS	
	September	4.4 ± 0.86		6.9 ± 0.04		
	July	4.6 ± 0.30	NS	7.0 ± 0.03	< 0.05*	
	September	4.4 ± 0.86		6.9 ± 0.04		
Treatment and host species	Control	4.2 ± 0.10	NS	7.2 ± 0.02	< 0.001***	Soybean and Corn
	NST	4.1 ± 0.08		7.0 ± 0.03		
	Control	4.4 ± 0.15	NS	7.2 ± 0.03	< 0.01**	Soybean
	NST	4.2 ± 0.10		7.1 ± 0.03		
	Control	4.0 ± 0.12	NS	7.1 ± 0.05	< 0.01**	Corn
	NST	3.9 ± 0.10		7.0 ± 0.05		
Treatment and month	Control	4.6 ± 0.30	NS	7.2 ± 0.03	< 0.001***	July
	NST	4.4 ± 0.20		7.0 ± 0.04		
	Control	3.8 ± 0.11	NS	7.2 ± 0.04	< 0.001***	August
	NST	3.8 ± 0.11		7.0 ± 0.05		
	Control	4.4 ± 0.09	NS	7.1 ± 0.05	NS	September
	NST	4.2 ± 0.08		7.1 ± 0.05		
Treatment and year	Control			7.1 ± 0.05	NS	2016
	NST			7.0 ± 0.05		
	Control			7.1 ± 0.05	< 0.01**	2017
	NST			6.9 ± 0.05		
	Control			7.2 ± 0.03	NS	2018
	NST			7.2 ± 0.03		

Variance in the bacterial alpha diversity (Shannon index) explained by year, month, neonicotinoid seed treatment (NST) in soybean and corn together and individually, interactions between NST and month, and interactions between NST and year. Means and standard errors (SE) of each group are calculated and compared. The significance of their differences is determined using a non-parametric Wilcoxon rank-sum test, and the *P*-values are adjusted using Holm’s method. Significance levels for each variable are given by: ****P < 0.0001; ***P < 0.001; **P < 0.01; *P < 0.05; NS: *P* ≥ 0.05.

### Effects of Neonicotinoid Seed Treatment on Bacterial Communities

**FIGURE 3:**
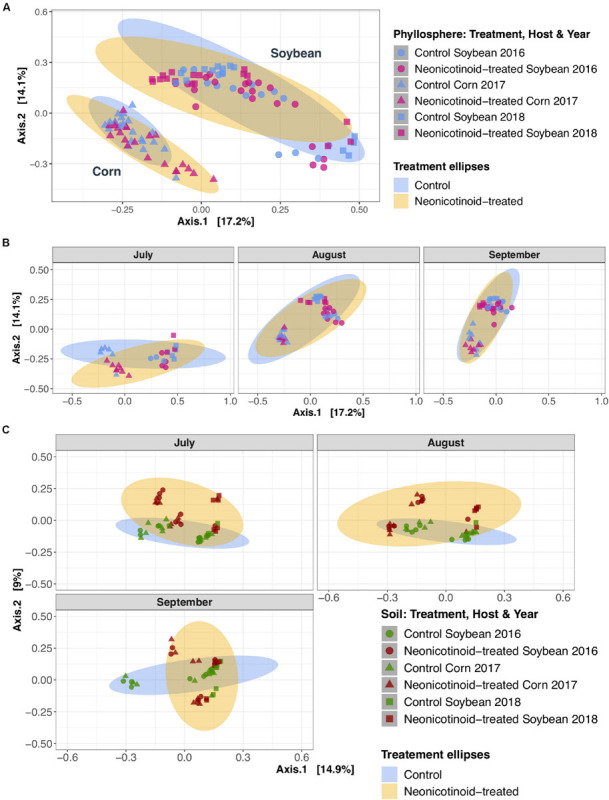
Phyllosphere and soil bacterial community composition variations in response to neonicotinoid seed treatment. Principal coordinate analysis (PCoA) on Bray-Curtis dissimilarities demonstrates the composition of phyllosphere **(A,B)** and soil **(C)** bacterial community in a 3-year soybean (2016: circles and 2018: cubes) and corn (2017: triangles) rotation in L’Acadie, Quebec, Canada. The phyllosphere bacterial community variation among control (blue points) and neonicotinoid-treated (pink points) samples is masked by the effects of host species **(A)** and time **(B)**. While in soil **(C)**, the bacterial communities vary among control (green points) and neonicotinoid-treated (red points) samples. Ellipses are shaded based on treatment (blue for control and yellow for neonicotinoid-treated samples) and represent a 95% confidence level.

**FIGURE 4:**
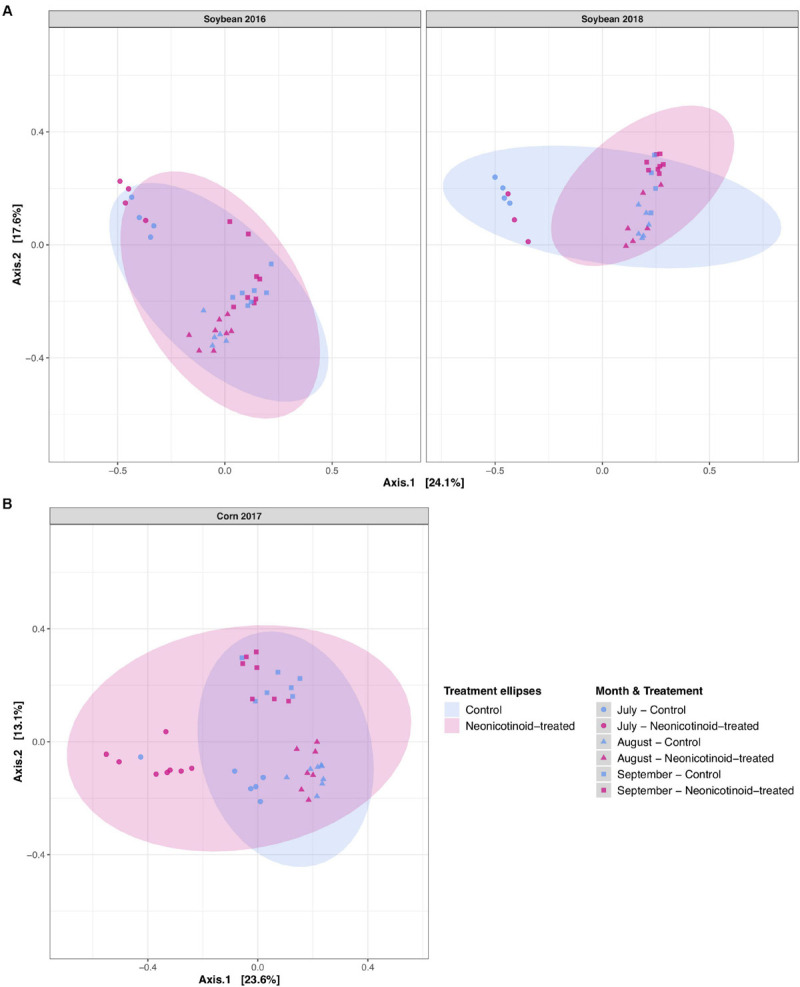
Soybean and corn phyllosphere bacterial community composition variations in response to neonicotinoid seed treatment and year. Principal coordinate analysis (PCoA) on Bray-Curtis dissimilarities illustrates the phyllosphere bacterial variation individually for each host species and year of rotation in L’Acadie, Quebec, Canada: **(A)** soybean (left: year 2016; right: year 2018) and **(B)** corn (year 2017). The shapes of the points represent the month and the colors show the treatment. Ellipses are shaded based on treatment (blue for control and pink for neonicotinoid-treated samples) and represent a 95% confidence level.

Neonicotinoid seed treatment showed complex effects on the composition of bacterial communities. Neonicotinoid treatment alone explained a small but significant portion of the variation in both the phyllosphere (1.3%) and soil (2.6%) (PERMANOVA *P* < 0.01, Table 3). Since the bacterial composition varied greatly among host species and time (Table 3 and Figure 3), the impacts of neonicotinoid seed treatment were partially masked by this variation. Effects of neonicotinoid treatment were especially evident in soils in the middle of the growing season (Figure 3C). To uncover neonicotinoid impacts, we analyzed each crop species separately, which revealed a much stronger effect of the neonicotinoid seed treatment on the composition of the phyllosphere communities in corn (5.3%) than in soybean (1.6%) (PERMANOVA *P* < 0.001 and *P* < 0.05, respectively, Table 3 and Figure 4). There was no significant difference in phyllosphere alpha diversity between neonicotinoid treatments overall, but soil bacterial alpha diversity was significantly higher in control versus neonicotinoid-treated samples (Shannon index mean ± SE 7.2 ± 0.02 versus 7.0 ± 0.03, Wilcoxon adjusted *P* < 0.001, Table 2).

**TABLE 3:** Drivers of the phyllosphere and soil bacterial community composition variation in response to neonicotinoid seed treatment in a 3-year soybean/corn rotation.

**Variables**	**Bray-Curtis dissimilarities**	**Phyllosphere**						**Soil**					
		**Soybean and corn**		**Soybean**		**Corn**		**Soybean and corn**		**Soybean**		**Corn**	
Host species	R^2^ (%)	14.7						2.4					
	*F* | Pr(>*F*)	32.9	0.001***					3.61	0.001***				
Year	R^2^ (%)	7.4		13.1				5.7		9.2			
	*F* | Pr(>*F*)	16.6	0.001***	16.8	0.001***			8.42	0.001***	8.92	0.001***		
Month	R^2^ (%)	15.2		28.3		30		2.6		3.2		5.8	
	*F* | Pr(>*F*)	16.96	0.001***	18.27	0.001***	9.25	0.001***	1.89	0.001***	1.55	0.012*	1.39	0.036*
NST	R^2^ (%)	1.3		1.6		5.3		2.6		3.4		3.7	
	*F* | Pr(>*F*)	2.81	0.002**	2.12	0.021*	3.3	0.001***	3.82	0.001***	3.33	0.001***	1.78	0.017*
NST: Host species	R^2^ (%)	1.2						NS					
	*F* | Pr(>*F*)	2.56	0.002**					NS	NS				
NST: Year	R^2^ (%)	0.8		NS				1.1		1.8			
	*F* | Pr(>*F*)	1.73	0.043*	NS	NS			1.63	0.030*	1.73	0.014*		
NST: Month	R^2^ (%)	NS		NS		NS		2.4		NS		5.6	
	*F* | Pr(>*F*)	NS	NS	NS	NS	NS	NS	1.8	0.002**	NS	NS	1.34	0.048*
NST: Month: Host species	R^2^ (%)	1.4						NS					
	*F* | Pr(>*F*)	1.55	0.028*					NS	NS				
NST: Year: Month	R^2^ (%)	1.4		NS				NS		NS			
	*F* | Pr(>*F*)	1.57	0.026*	NS	NS			NS	NS	NS	NS		

PERMANOVA (Bray-Curtis dissimilarities) identifies the proportion of bacterial community composition variation explained by host plant species, time (year and month), neonicotinoid seed treatment (NST) and their interactions in the soybean and corn phyllosphere and soil in response to neonicotinoid seed treatment in a 3-year soybean/corn rotation in L’Acadie, Quebec, Canada. (:) represents the interaction between variables. Significance levels for each variable are given by: ***P < 0.001; **P < 0.01; *P < 0.05; NS, *P* ≥ 0.05.

The overall effect of neonicotinoid seed treatment on the temporal variation of bacterial community composition and alpha diversity was weak. In the phyllosphere, although there was a small significant effect of the interaction between neonicotinoid application and time (month and year) on variation in community composition (1.4%, PERMANOVA *P* < 0.05, Table 3), the impacts on inter-annual variation and specific interactions with individual host species were not significant. The interaction of neonicotinoid seed treatment and time was slightly stronger in soil, especially with month (2.4%, PERMANOVA *P* < 0.01, Table 3). Uncovering these effects by studying each crop separately revealed that this month-to-month temporal variation in bacterial community structure within a growing season was particularly important in corn (5.6%, PERMANOVA *P* < 0.05, Table 3). Similarly, while the interaction between neonicotinoid seed treatment with time had no significant effect on bacterial alpha diversity in the phyllosphere, soil alpha diversity was significantly reduced in the neonicotinoid-treated samples in July and August (interaction between neonicotinoid seed treatment and month: linear regression analysis of Shannon index, *F* = 6.27, ANOVA *P* < 0.001; significant interactions among months and treatment: Shannon index, Wilcoxon *P* < 0.001, Table 2).

### Bacterial Taxa Impacted by Neonicotinoid Seed Treatment

**FIGURE 5:**
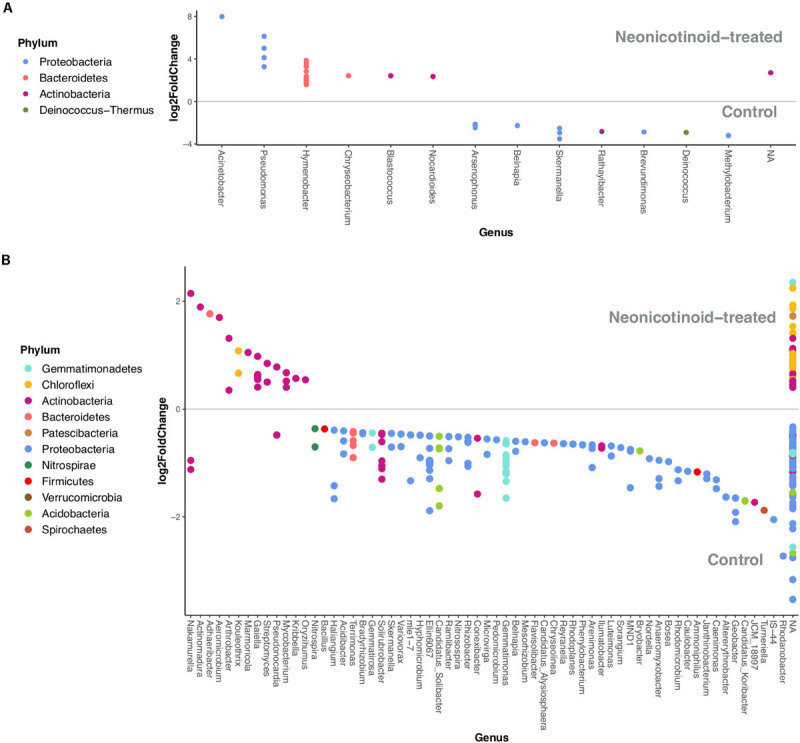
Phyllosphere and soil bacterial taxa (phyla and genera) associated with control and neonicotinoid seed treatment. Differential expression analysis of sequence data (DESeq2) illustrates the bacterial ASVs that are significantly differentially abundant (adjusted *P* < 0.05) between control and neonicotinoid-treated samples of soybean and corn phyllosphere **(A)** and soil **(B)** in a 3-year rotation in L’Acadie. Each point represents one ASV related to a genus on the x-axis, and its color shows the phylum it belongs to. The ASVs on the top of each graph (log2FoldChange > 0) are associated with the neonicotinoid-treated samples, while the others (log2FoldChange < 0) are related to the controls.

Neonicotinoid seed treatment led to changes in the relative abundance of some phyllosphere and soil bacterial ASVs. Overall, we detected 34 bacterial ASVs in the phyllosphere and 294 in soil that were significantly differentially abundant between the control and neonicotinoid-treated samples. In the phyllosphere, 22 ASVs (mainly Bacteroidetes) were more abundant, and 12 (mainly Proteobacteria) were less abundant in response to neonicotinoid seed treatment (Table 4). The genera *Hymenobacter* (13 ASVs) and *Pseudomonas* (4 ASVs) were particularly favored by neonicotinoid treatment, while the genera *Arsenophonus* (4 ASVs) and *Skermanella* (3 ASVs) among others decreased in abundance in neonicotinoid-treated samples (DESeq2 adjusted *P* < 0.05, Figure 5A and Supplementary Table 3). In soil, 68 ASVs (mainly Actinobacteria and Chloroflexi) were more abundant in the neonicotinoid-treated samples, while 226 (mainly Proteobacteria) were less abundant (Table 4). More than 60 genera of soil bacteria were significantly impacted by neonicotinoid treatment (Figure 5B and Supplementary Table 4). Genera negatively affected by neonicotinoid treatment included some of the beneficial soil bacteria (e.g., *Ammoniphilus*, *Bacillus*, *Bosea*, *Bradyrhizobium*, *Hyphomicrobium*, *Mesorhizobium*, *Microvirga*, *Nitrospira*, *Nitrosospira*, *Rhizobacter*, and *Rhodanobacter*) while the genera favored by the neonicotinoid treatment were dominated by Actinobacteria, including genera potentially involved in neonicotinoid degradation [e.g., *Mycobacterium* (Kandil et al., 2015) and *Streptomyces* (Guo et al., 2019)] or other pesticides degradation [e.g., *Arthrobacter* (Tam et al., 1987)].

**TABLE 4:** Phyllosphere and soil bacterial phyla associated with control and neonicotinoid seed treatment.

**Habitat**	**Phylum**	**Number of ASVs associated with treatment**	
		**Control**	**Neonicotinoid-treated**
Phyllosphere	Actinobacteria	1	3
	Bacteroidetes	0	14
	Deinococcus-Thermus	1	0
	Proteobacteria	10	5
Soil	Acidobacteria	11	0
	Actinobacteria	27	31
	Bacteroidetes	11	1
	Chloroflexi	3	33
	Firmicutes	2	0
	Gemmatimonadetes	27	2
	Nitrospirae	2	0
	Patescibacteria	0	1
	Proteobacteria	139	0
	Spirochetes	1	0
	Verrucomicrobia	3	0

*Differential expression analysis of sequence data (DESeq2) identified the bacterial phyla of the ASVs that are significantly differentially abundant (adjusted *P* < 0.05) between control and neonicotinoid-treated samples of soybean and corn phyllosphere and soil in a 3-year rotation in L’Acadie.*

## Discussion

Our findings indicate that habitat (soil versus phyllosphere), host species (soy versus corn), time, and their interactions are all strong drivers of bacterial composition variation in a soybean and corn agroecosystem. While this result is perhaps not surprising given that previous studies have identified these factors as important drivers of phyllosphere (Knief et al., 2010; Kembel et al., 2014; Laforest-Lapointe et al., 2016a) and soil bacterial communities (Wieland et al., 2001; Tarlera et al., 2008; Hannula et al., 2019), our results suggest that complex interactions among these factors drive overall community composition and diversity. In particular, we have shown a role for temporal variation, alone and in interaction with habitat and host species, as an important driver of bacterial community composition variation, especially in the phyllosphere. While succession of microbial communities in the phyllosphere has been documented previously (Redford and Fierer, 2009; Wagner et al., 2016; Manching et al., 2017), here we have shown that even in a rotation of annual crops, the patterns of bacterial succession within and among years are an important driver of community structure.

We have shown that neonicotinoid seed treatments have a non-target impact on bacterial community structure and diversity in a soybean/corn agroecosystem, in particular on the taxonomic composition of soil bacterial communities over the growing season. Phyllosphere and soil bacteria exhibit different patterns of community composition, alpha diversity and temporal variation throughout the growing season and in response to neonicotinoid application. In the phyllosphere, host plant species and time are stronger drivers of bacterial community variation than neonicotinoid seed treatment; however, neonicotinoids interact with these parameters to influence the phyllosphere bacterial community composition. Overall, soil bacteria exhibited stronger changes in community composition and a significant decline in bacterial alpha diversity in response to neonicotinoid treatment, while phyllosphere bacteria responses to neonicotinoids were weaker. Our results complement previous lab-based studies of neonicotinoid effects on bacterial communities (Cai et al., 2016; Zhang et al., 2018; Yu et al., 2020), providing some of the first field-based evidence that neonicotinoids impact bacterial diversity in agroecosystems.

Overall, soil bacterial communities were more affected by neonicotinoid pesticide treatment than phyllosphere bacterial communities. Neonicotinoid effects on soil bacterial community composition and diversity varied greatly in time, with the impacts of neonicotinoid application on the soil bacterial community composition and alpha diversity most pronounced in the middle of the growing season. We suggest that this could be explained by the fact that neonicotinoids’ active period is much shorter in plants (Myers and Hill, 2014; Alford and Krupke, 2017) than in soils, where they potentially persist for months or years (Goulson, 2013; Bonmatin et al., 2015). Despite the reported accumulation potential of neonicotinoids in soils over time (Wood and Goulson, 2017), we did not observe any significant inter-annual difference in bacterial diversity among years in interaction with the pesticide treatment, perhaps due to degradation or leaching of the neonicotinoids (Banerjee et al., 2008; Kurwadkar et al., 2013).

We also observed that the more homogenous the bacterial community composition is, the more it is altered by the neonicotinoid application (soil more than phyllosphere and corn phyllosphere more than soybean phyllosphere). We need further studies to determine if the homogeneity of the bacterial communities resulted in less resilience in response to perturbations or if less variability within groups allowed us to notice more changes in the communities.

In addition to community-wide responses of bacteria to the neonicotinoid treatment, numerous bacterial taxa increased or decreased in relative abundance in response to neonicotinoids. Bacterial taxa that were favored by the pesticide treatment include several genera that are known to be potentially involved in neonicotinoid degradation [e.g., *Hymenobacter* (Guo et al., 2020), *Mycobacterium* (Kandil et al., 2015), *Pseudomonas* (Pandey et al., 2009), and *Streptomyces* (Guo et al., 2019)]. In soils, there was a decline in the relative abundance of several ASVs from Proteobacteria and Gemmatimonadetes phyla and an increase in some ASVs from Chloroflexi and Actinobacteria, a result partially in accordance with a previous study that reported a decrease in the relative abundance of Gemmatimonadetes and OD1 phyla and an increase in the relative abundance of the Chloroflexi and Nitrospirae phyla in response to the neonicotinoid treatments (Yu et al., 2020).

Neonicotinoid seed treatment led to decreases in the relative abundance of several potentially beneficial soil bacteria, including the plant growth-promoting rhizobacteria (PGPR) that are capable of developing a symbiotic association with host plants [e.g., *Bacillus, Bosea, Mesorhizobium*, and *Rhizobacter* (Podile and Krishna Kishore, 2006)], nitrogen-fixing bacteria [e.g., *Bradyrhizobium* and *Microvirga* (Kumar et al., 2015)], and other bacteria involved in the nitrogen cycle [e.g., *Ammoniphilus, Hyphomicrobium, Nitrospira, Nitrosospira* and *Rhodanobacter* (Pitombo et al., 2016)]. While plant growth and yield variations in response to the pesticide application were not determined in our research, a recent study conducted in the same bioclimatic conditions indicated no significant impact on yield in the absence of the targeted pests (Labrie et al., 2020). However, although we did not measure the effects of neonicotinoid treatments on ecosystem processes such as nitrification, our results suggest a potential mechanism for the negative effects of neonicotinoids on nitrification that have been observed in previous studies (Filimon et al., 2015; Zhang et al., 2018).

Given that invertebrates are the main target of neonicotinoids, we suggest that the effects of this pesticide on bacterial communities could be related to the trophic interactions between bacteria and the invertebrates (e.g., free-living nematodes and microarthropods) affected by neonicotinoids. This insecticide may indirectly alter the bacterial community composition by affecting the top-down regulation of these communities through reducing the higher trophic levels that feed on bacteria (Staley et al., 2015; Thakur and Geisen, 2019). Future research to evaluate the effects of neonicotinoids on these eukaryotic microbial communities, the trans-kingdom and trophic interactions between them and bacterial communities, and especially the prey-predator dynamics, as well as gene expression and functional variations of microbial communities, will improve our understanding of the mechanisms driving the microbial community variations in response to the pesticide application.

## Conclusion

To date, there have been few studies that have evaluated the impacts of neonicotinoid seed treatments on phyllosphere and soil bacterial communities. To our knowledge, this study is the first with an experimental design that represents real farming conditions in a crop rotation. Despite the fact that neonicotinoids target invertebrates, we observed their non-target impacts on bacterial communities of the phyllosphere and soil, especially the beneficial bacteria that are crucial for plant growth and health and soil fertility and quality. Future studies to identify the genomic and physiological features associated with tolerance of neonicotinoids will be required to understand the mechanistic reasons for these associations. Investigating the biological and trophic interactions among bacteria and other micro- and macro-organisms that are affected by pesticides will help us to better understand the non-target effects of pesticides on microbial diversity and how to control them with better agricultural practices.

## Data Availability Statement

We have deposited the raw sequences at the NCBI Sequence Read Archive (SRA accession number PRJNA662376). Scripts to perform the analyses of the current study are available in the following GitHub repository: https://github.com/memoll/acadie_16s.

## Author Contributions

MP, BM, and SK conceived and designed the study. BM and SK obtained the funding. MP collected and analyzed the data and wrote the manuscript. All authors critically reviewed and edited the manuscript.

## Conflict of Interest

The authors declare that the research was conducted in the absence of any commercial or financial relationships that could be construed as a potential conflict of interest.

## Footnotes

1 http://jgi.doe.gov/data-and-tools/bb-tools/

## Supplementary Material

Supplementary Table 1 Soil physicochemical properties. For each plot, physicochemical properties of soil were measured every year, twice (2016 and 2017) or three times (2018) during the growing seasons.

Supplementary Table 2 Correlation between bacterial families relative abundances and their habitats and host species. Envfit analysis of correlations between bacterial families of soybean and corn phyllosphere and soil with an average relative abundance of more than 0.01 and the axes of PCoA ordination (on Bray-Curtis dissimilarities). (✓) and (−), respectively, show whether the bacterial family is significantly correlated with the soybean or corn phyllosphere or soil or not. Significance levels for each variable are given by: ^∗∗∗^*P* < 0.001; ^∗∗^*P* < 0.01; ^∗^*P* < 0.05; NS: *P* ≥ 0.05.

Supplementary Table 3 Significantly differentially bacterial ASVs of phyllosphere associated with control and neonicotinoid seed treatment. Differential expression analysis on sequence data (DESeq2) detects the bacterial ASVs that are significantly differentially abundant (adjusted *P* < 0.05) between soybean and corn phyllosphere control and neonicotinoid-treated samples in a 3-year rotation in L’Acadie. The ASVs with a positive log2FoldChange are associated with the neonicotinoid-treated samples, while the ones with a negative log2FoldChange are related to the controls.

Supplementary Table 4 Significantly differentially soil bacterial ASVs associated with control and neonicotinoid seed treatment. Differential expression analysis on sequence data (DESeq2) identifies the ASVs that are significantly differentially abundant (adjusted *P* < 0.05) between the control and neonicotinoid-treated samples of soybean and corn soil bacteria in a 3-year rotation in L’Acadie. The ASVs favored by neonicotinoid-treated samples have a positive log2FoldChange, while the ASVs associated with control have a negative log2FoldChange.

## Abbreviations

ANOVA: Analysis of variance
ASV: Amplicon sequence variant
DESeq2: Differential expression analysis of sequence data
FDR: False-discovery rate
NMDS: Non-metric multidimensional scaling
NST: Neonicotinoid seed treatment
PCoA: Principal coordinate analysis
PERMANOVA: Permutational analysis of variance
SE: Standard error.
